# Prioritization of disease microRNAs through a human phenome-microRNAome network

**DOI:** 10.1186/1752-0509-4-S1-S2

**Published:** 2010-05-28

**Authors:** Qinghua Jiang, Yangyang Hao, Guohua Wang, Liran Juan, Tianjiao Zhang, Mingxiang Teng, Yunlong Liu, Yadong Wang

**Affiliations:** 1 Center for Biomedical Informatics, School of Computer Science and Technology, Harbin Institute of Technology, Harbin, Heilongjiang, 150001, China; 2 Center for Computational Biology and Bioinformatics, Indiana University School of Medicine, Indianapolis, IN 46202, USA

## Abstract

**Background:**

The identification of disease-related microRNAs is vital for understanding the pathogenesis of diseases at the molecular level, and is critical for designing specific molecular tools for diagnosis, treatment and prevention. Experimental identification of disease-related microRNAs poses considerable difficulties. Computational analysis of microRNA-disease associations is an important complementary means for prioritizing microRNAs for further experimental examination.

**Results:**

Herein, we devised a computational model to infer potential microRNA-disease associations by prioritizing the entire human microRNAome for diseases of interest. We tested the model on 270 known experimentally verified microRNA-disease associations and achieved an area under the ROC curve of 75.80%. Moreover, we demonstrated that the model is applicable to diseases with which no known microRNAs are associated. The microRNAome-wide prioritization of microRNAs for 1,599 disease phenotypes is publicly released to facilitate future identification of disease-related microRNAs.

**Conclusions:**

We presented a network-based approach that can infer potential microRNA-disease associations and drive testable hypotheses for the experimental efforts to identify the roles of microRNAs in human diseases.

## Background

The identification of genes associated with human diseases is an important goal of biomedical research. Recently, a number of computational methods have been developed to predict or prioritize disease-related protein-coding genes [1-23]. Most approaches are based on the idea that dysfunctions of functionally related protein-coding genes tend to be associated with phenotypically similar diseases [1,2,6,12,13,15,16,18-22]. These protein-coding genes linked to similar diseases usually interact with each other or participate in the common biological modules. Network-based approaches have also been employed to predict or prioritize new candidate disease genes based upon network linkages with known disease genes [1-3,12,23]. These approaches typically start with constructing a gene-gene association network based on one or more types of genomic and proteomic information, and then prioritize candidate protein-coding genes based on network proximity to known disease-related genes. For example, Franke *et al*. and Linghu *et al*. separately constructed a functional linkage network (FLN) by integrating multiple types of data, such as protein-protein interaction, microarray and Gene Ontology annotation data, and utilized the FLN for disease gene prioritization [7,23]. Lage *et al.* constructed a human phenome-interactome network and scored each candidate protein based on the involvement of its direct network neighbors in similar diseases [1]. The biological interpretation of a high-scoring candidate was that the candidate was likely to be involved in the molecular mechanism of the disorder of interest.

Growing evidence indicates that microRNAs play important roles in the development and progression of human diseases, and are critical for the prognosis, diagnosis and the evaluation of treatment responses for these diseases [24-37]. Jiang *et al.* and Lu *et al.* independently developed two manually curated database--miR2Disease [38] and Human MicroRNA Disease Database (HMDD) [39], which aim at providing a comprehensive resource of experimentally verified microRNA-disease associations. However, one major issue in microRNA studies is the lack of bioinformatics methods to infer potential microRNA-disease associations that can guide further biological experiments.

MicroRNAs exert their biological functions through suppression of their target genes [40]. Accumulating studies indicate that microRNAs usually perform related functions by targeting either the same genes or functionally related genes in a coordinated manner [34,35,41-48]. It has become an increasingly important and informative approach to analyze biological systems and disease mechanisms in networks of genes and diseases [6,12,21,49,50]. Establishing a functional relationship between two microRNAs by their target genes and further constructing a functionally related microRNA network will be useful for understanding the roles of microRNAs in diseased states.

**Figure 1:**
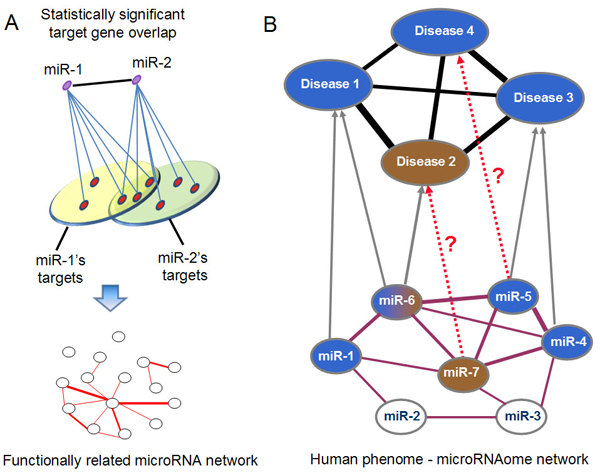
**Construction and application of a human phenome-microRNAome network.** (A) Construction of a functionally related microRNA network. An edge is placed between two microRNAs if they share significant number of target genes. (B) Application of the phenome-microRNAome network to infer new microRNA-disease associations. A gray edge connects known disease-related microRNA to the corresponding disease. Disease 2 has a related microRNA (miR-6), and disease 4 doesn’t have any related microRNAs. The red dash lines represent the potential microRNA-disease associations that might be predicted by this network model.

Herein, we propose a computational approach to infer potential microRNA-disease associations by prioritizing the entire human microRNAome for diseases of interest. It was a logical extension of previous network-based method for predicting or prioritizing disease-related protein-coding genes. We first constructed a functionally related microRNA network (Figure 1A ) and a human phenome-microRNAome network (Figure 1B). We subsequently examined whether functionally related microRNAs tend to be associated with phenotypically similar diseases and prioritized microRNAs for human diseases.

## Results

### Construction of human phenome-microRNAome network

In order to prioritize the entire microRNAome for diseases, we constructed a functionally related microRNA network by assuming that two microRNAs are functionally related if the overlap between their target genes was statistically significant (Figure 1A). A *p*-value from Fisher’s Exact Test was used to evaluate the overlap, and was adjusted by the Benjamini-Hochberg correction [51,52]. Two microRNAs were considered to be functionally related if the adjusted *p*-value was less than 0.001. Following this strategy, and using microRNA-target dataset predicted by Probability of Interaction by Target Accessibility (PITA) [53], we obtained a functionally related microRNA network that included 9,249 relationships (edges) between 514 microRNAs (nodes). We subsequently constructed a hypothetical human phenome-microRNAome network by integrating the microRNA network with a phenome network [1,12,20] using 270 experimentally verified microRNA-disease associations (Figure 1B).

### Functionally related microRNAs tend to be associated with phenotypically similar diseases

Our model was based on the notion that functionally related microRNAs tend to be associated with phenotypically similar diseases. We examined it by addressing two questions: (1) whether disease pairs associated with common microRNAs are phenotypically more similar, as opposed to randomly selected phenotype pairs; and (2) whether the microRNA pairs associated with common diseases are functionally more related. Because we have constructed a functionally related microRNA network, the functional relatedness between two microRNAs can be measured through the number of shared network neighbors and the length of the shortest path in the microRNA network. We chose to use these two measures mainly based on the standpoint that, in a functional network, if two nodes are less distant from each other or share more neighbors, they are functionally more related. Herein, we used the function *e ^-x^* to convert the length of the shortest path to the degree of functional relatedness between two microRNAs.

**Figure 2:**
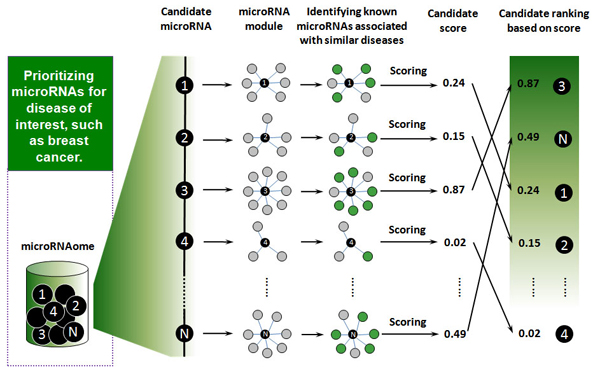
**Functionally related microRNAs tend to be associated with Phenotypically similar diseases.** (A) The observed average phenotypic similarity score (arrow) of 349 phenotype pairs associated with common microRNAs and the distribution of expected average phenotypic similarity scores (curve) of 10,000 random control sets containing the same number of randomly sampled phenotype pairs (p<10^-4^). (B, C) The observed average functional relatedness (arrow) of 1,252 microRNA pairs associated with common diseases and the distribution of the expected average functional relatedness (curve) of 10,000 random control sets containing the same number of randomly sampled microRNA pairs (p<10^-4^). The measures for functional relatedness between microRNAs are the average number of shared network neighbors and a function value that is derived from the shortest path length.

A total of 349 disease pairs were identified to be associated with common microRNAs, and 1,252 microRNA pairs were found to be associated with common diseases. To evaluate the statistical significance of the phenotypic similarity between diseases associated with common microRNAs, we generated 10,000 negative control sets and calculated an average phenotypic similarity score for each set containing 349 disease pairs that were randomly sampled from the human phenome. The average phenotypic similarity score between diseases associated with common microRNAs was significantly higher than the similarity of randomly selected phenotype pairs from the human phenome (*p* < 10^-4^, Figure 2A). In a similar manner, we generated another 10,000 negative control sets and calculated the average functional relatedness for each set containing 1,252 microRNA pairs randomly sampled from the microRNA network. The microRNA pairs associated with common diseases shared more common network neighbors (*p* < 10^-4^, Figure 2B), and were less distant from each other in the microRNA network (*p* < 10^-4^, Figure 2C).

### Performance evaluation

In order to assess the power of our model to infer microRNA-disease associations by prioritizing the entire microRNAome, we performed the leave-one-out cross-validation on 270 known experimentally verified microRNA-disease associations. Each association was left out once as the testing case, being referred to as *<m, d>*. For the disease *d*, the microRNA *m* was termed ‘defector’ microRNA. We prioritized the entire microRNAome according to the scores derived from the scoring system. Note that the score can be computed only for all microRNAs in the microRNA network, which was termed the ranked microRNAome. If the ranking of the ‘defector’ microRNA exceeds a given threshold, the model successfully predicts the experimentally verified association *<m, d>*.

**Figure 3:**
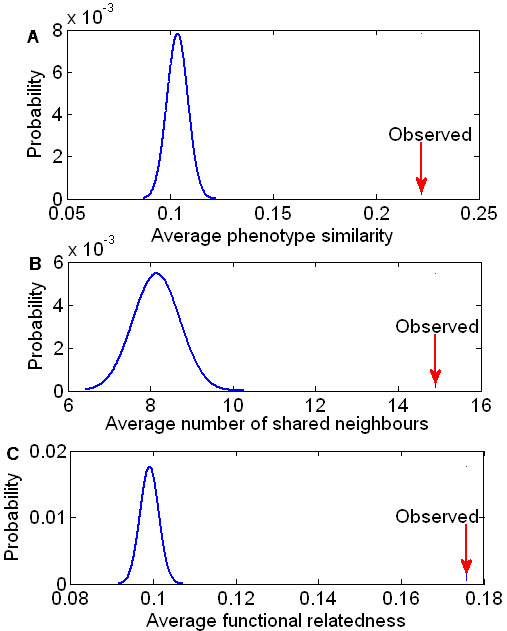
**Leave-one-out cross-validation results.** The red curve was derived from 270 experimentally verified microRNA-disease associations. The blue curve represents the performance of the model to prioritize microRNAs for diseases with which no microRNAs have been experimentally verified to be associated. The green curve was obtained from 270 randomly generated microRNA-disease associations.

We calculated the sensitivity and specificity for each threshold. Sensitivity refers to the percentage of the ‘defector’ microRNAs whose ranking is higher than a given threshold, namely the ratio of the successfully predicted experimentally verified microRNA-disease associations to the total experimentally verified microRNA-disease associations. Specificity refers to the percentage of microRNAs that are below the threshold. The same computational strategies were applied by Endeavour [13] and GeneWanderer [2]. A receiver-operating characteristics (ROC) curve was plotted by varying the threshold, and the standard area under curve (AUC) was calculated. When our model was tested on 270 experimentally verified microRNA-disease associations, an AUC of 75.80% was achieved (red curve in Figure 3), suggesting that our model can recover the known experimentally verified microRNA-disease associations, and therefore has the potential to infer new microRNA-disease associations by prioritizing the microRNAome.

In order to ensure that the prioritization represents biological significance, the model was tested on the 270 randomly generated microRNA-disease associations, which resulted in an AUC of 49.81% (green curve in Figure 3), approximate to the uninformative AUC of 50% (dash line in Figure 3). This result showed indirectly that our model can obtain a biologically meaningful prioritization.

### Applicability of the model to diseases without any known related microRNAs

To demonstrate that our model is applicable to the diseases without any known related microRNAs, we removed all other experimentally verified microRNA-disease associations that are involved in the disease *d*, for each of the 270 known experimentally verified microRNA-disease associations, denoted as *<m, d>*. This step ensured that prioritizing microRNAs for the disease *d* only took advantage of the information of other diseases having similar phenotypes with the disease *d*. When our model was tested on this dataset, an AUC of 69.51% was obtained (blue curve in Figure 3), suggesting that the model had the potential to achieve the goal of predicting potential microRNA-disease associations for the diseases without any known related microRNAs.

### Effect of microRNA families and robustness

MicroRNAs belonging to the same family have similar target profiles because they share the “seed” region close to the 5’ end of the microRNAs, which is the main determinant of microRNA targeting. One possible concern is the potential confounding effect of microRNA families in the performance evaluation procedure. If several microRNAs (*m_i_*, *i=1, 2*…) belonging to the same family are associated with a certain disease *d*, it might be relatively easy for the leave-one-out cross-validation procedure to recover the experimentally verified microRNA-disease association *<m_i,_ d>* being examined. To assess the possible effect of this confounding factor, we removed all other experimentally verified associations between the disease *d* and microRNAs which belong to the same family as the microRNA *m*. Following this procedure, a slightly reduced AUC of 71.39% was achieved (black curve in Figure 3), suggesting that microRNA families are not a main factor leading to the good performance of our model.

There is great difference among the microRNA-target lists predicted by different algorithms. Herein, we examined the robustness of our model to another microRNA-target list predicted by TargetScan [54], one of the leading target prediction tools. We constructed another functionally related microRNA network by retrieving the 15,000 most significant microRNA-microRNA relationships (edges) between 541 microRNAs (nodes). Based on this microRNA network, we created another phenome-microRNAome network and obtained a comparable performance by testing the model on the 270 known microRNA-disease associations, indicating that our model isn’t limited to a specific target prediction algorithm.

### Prioritizing the entire microRNAome for 1,599 disease phenotypes

Many disease microRNAs have been identified over the past decade. However, the majority of diseases in the OMIM database aren’t associated with any microRNA. One reason is that no sufficient efforts have been made to decipher potential roles of microRNAs in those diseases. To provide testable hypotheses to guide future experiments, it is important to computationally infer possible microRNA-disease associations for diseases of interest.

Two disease phenotypes were defined to be similar if they have a phenotypic similarity score no less than 0.3 [19]. We thus obtained 1,599 disease phenotypes, which are similar to at least one of the disease phenotypes in the benchmark dataset (see Additional file 1). We prioritized the entire microRNAome for 1,599 disease phenotypes according to score derived from the scoring system. In addition, the top 100 microRNAs for each of the 1,599 phenotypes are publicly released to facilitate the discovery of disease microRNAs (see Additional file 2).

### Case study: breast cancer

We presented a case study for breast cancer, which is one of the most commonly occurring cancers among women and accounts for 22% of all female cancers. We prioritized all microRNAs for breast cancer. Among the top 100 microRNAs, 17 have been confirmed to contribute to the development of breast cancer, and 13 were verified to be deregulated in breast cancer cells. By literature retrieval, we provided more supporting evidence in Additional file 3. For example, Reddy *et al*. found that miR-7 inhibits p21-activated kinase 1 (Pak1) expression, a widely up-regulated signaling kinase in multiple human cancers, and the miR-7 introduction inhibits the motility, invasiveness, anchorage-independent growth and tumorigenic potential of highly invasive breast cancer cells [55]. Foekens *et al*. also linked miR-7 to breast cancer aggressiveness [56]. In addition, Scott *et al*. found that miR-125b is down-regulated in breast cancer and miR-125a or miR-125b-overexpressing SKBR3 cells displayed diminished plating and anchorage-dependent growth in addition to markedly reduced cell migration and invasion capacities.

## Discussion

We demonstrated that the method we proposed achieved good performance in recovering known, experimentally verified microRNA-disease associations. Using the model, we prioritized the entire microRNAome for 1,599 diseases, most of which have not been linked to any microRNAs. The power of our model can be attributed to several factors. First, we constructed a functionally related microRNA network, which can capture the biological characteristics of some microRNAs that tend to exert the same or similar functions by the inhibition of common target genes in a coordinated manner. Second, we took full advantage of large-scale phenotype similarity score information, whose significance has been confirmed in several previous studies aiming at the identification of disease-related protein-coding genes [1,12]. Third, we used experimentally verified microRNA-disease associations, which allow connecting the human disease network with the microRNA network, and therefore provide underlying knowledge for the role of microRNA in disease pathogenesis.

There are several potential limitations. First, the known experimentally verified microRNA-disease associations were insufficient. Second, the functionally related microRNA network was constructed based on the standpoint that two microRNAs are functionally related if the number of shared target genes is statistically significant. In reality, two microRNAs may be functionally related when their target genes reside in the same cellular pathways or functional modules [43,57], rather than overlap significantly. Therefore, integrating other bioinformatics sources such as Gene Ontology annotation and protein-protein interaction network data might improve model performance. In addition, modeling rules connecting phenotype with microRNA network may represent an important step on the path of the emerging field of “network medicine” [21,58].

## Conclusions

Evidence continually reinforces the notion that functionally related protein-coding genes are usually associated with phenotypically similar diseases. Based on this notion, many innovative methodologies have been proposed to predict or prioritize protein-coding genes for complex diseases [1-3,7,11,12]. In this study, we studied the functional correlation of microRNAs and found that disease pairs associated with common microRNAs were phenotypically more similar, and the microRNA pairs linked to common diseases were functionally more related. We further constructed an integrated phenome-microRNAome network, through which we devised a method that can recover the known experimentally verified microRNA-disease associations and prioritize the entire microRNAome for 1,599 diseases. The top 100 microRNAs for each of the 1,599 diseases are released publicly, which will provide testable hypotheses to guide further experiments and contribute to the identification of true disease-related microRNAs.

## Methods

### Data sources

We downloaded the disease phenotype similarity scores from the MimMiner [19], developed by Driel* et al.* who computed a phenotype similarity score for each phenotype pair by the text mining analysis of their phenotype descriptions in the Online Mendelian Inheritance in Man (OMIM) database [59]. The disease phenotype network was constructed based on the similarity score. Two phenotypes were considered to be similar and were linked by an edge if their similarity score was no less than 0.3. The similarity score is equal to 1 if two phenotypes are identical. The phenotypic similarity score has been successfully used to predict or prioritize disease-related protein-coding genes [3,12].

PITA [53] is a leading microRNA target prediction approach that considers multiple factors, such as seed pairing, site number, overall predicted pairing stability and predicted site accessibility. We downloaded the PITA target catalog version 6 (3/15 flank ALL 31-Aug-08) and retrieved 145,872 predicted associations between 670 microRNAs and 14,826 target genes with a score less than -10.0, a threshold suggested by PITA, In addition, we downloaded 205,587 associations between 675 microRNAs and 11,758 target genes predicted by TargetScan (version5.1, conserved sites) [54], another leading target prediction algorithm.

miR2Disease [38] and HMDD [39] databases provide comprehensive resources for microRNA deregulation in human disease. From these databases, we selected 270 high-quality experimentally verified microRNA-disease associations that microRNA deregulation has been experimentally verified to contribute to the disease development. For instance, Ma *et al*. reported that highly expressed miR-10b initiates tumor invasion and metastasis in breast cancer through translational inhibition of HOXD10, and eventually increases expression of RHOC, a pro-metastatic gene [60]. The 270 associations (see Additional file 1) were used as the benchmark dataset for the performance evaluation of our model.

microRNA family dataset was retrieved from miRBase database[61].

### Computational model

**Figure 4:**
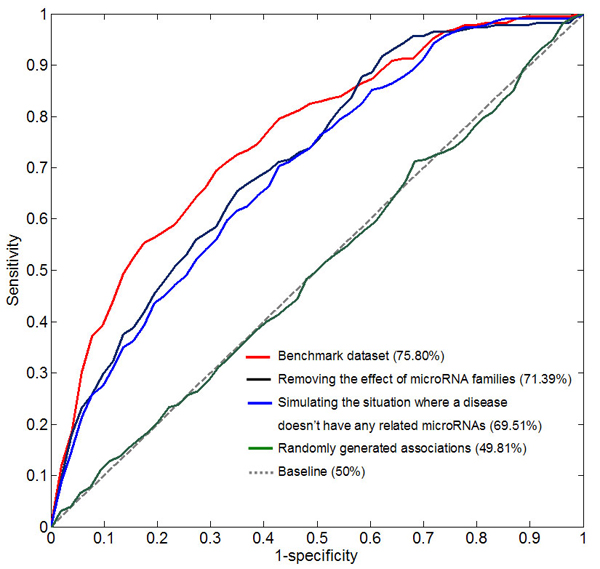
**Steps in prioritizing the entire microRNAome for a disease of interest.** First, a virtual pull-down of each candidate generates a hypothetical microRNA module, defined as a given microRNA (the center of the module) plus its direct network neighbors in the functionally related microRNA network. Second, in each microRNA module, the microRNAs linked to diseases that have similar phenotypes with the disease being investigated are identified. Finally, all candidates are scored and prioritized.

Based on the idea that functionally related microRNAs tend to be associated with phenotypically similar diseases, we developed a scoring system to assess how likely a microRNA may be involved in a specific disease phenotype. For a given disease *d*, a microRNA may be related if it and its direct network neighbors in the microRNA network contain microRNAs having been linked to the phenotypically similar diseases. All microRNAs were prioritized according to score. The top-ranked microRNAs can be expected to have a high probability of representing *bona fide* disease microRNAs, which will generate testable hypotheses to guide the future experiments and may significantly reduce the cost and effort to identify the *bona fide* disease microRNAs. The key steps are illustrated in Figure 4.

### Scoring system

The hypergeometric distribution is a discrete probability distribution that describes the number of successes in a sequence of *n* draws from a finite population without replacement. For example, there is a shipment of *N* objects in which *M* are defective. The hypergeometric distribution describes the probability that exactly *m* objects are defective in a sample of *n* distinct objects drawn from the shipment.

Herein, for a disease *d* of interest, each microRNA in the microRNA network is scored through the cumulative hypergeometric distribution:

The biological interpretation of a high-scoring microRNA is that it is likely to be involved in the disease *d*. Here, *N* is the total number of microRNAs in the whole functionally related microRNA network. *M* is the number of microRNAs in the whole microRNA network associated with diseases that are similar to the disease *d*. *n* denotes the number of microRNAs in the corresponding microRNA module. A module is defined as a given microRNA (the center of the module) plus its direct network neighbors. *m* is the number of microRNAs that are associated with similar diseases and are found in the corresponding module.

## Competing interests

The authors declare that they have no competing interests

## Authors' contributions

YW and YL conceived and designed the experiments. QJ, YH, GW, TZ and MT performed the experiments and analyzed the data. QJ, YW and YL wrote the paper.

## Supplementary Material

Additional file 1 Each line represents an association between a microRNA and a disease. Click here for file

Additional file 2 Each line represents a potential association between microRNA and disease, including MIM ID, microRNA ID and score. Click here for file

Additional file 3 Click here for file
