# Supplementary material for: Prioritization of disease microRNAs through a human phenome-microRNAome network
Source: BMC Syst Biol. 2010 May 28;4(Suppl 1):S2. doi: 10.1186/1752-0509-4-S1-S2 (PMC2880408; doi:10.1186/1752-0509-4-S1-S2)
Supplement: Additional file 3 [file 1752-0509-4-S1-S2-S3.doc]

| **Supplementary Table 1.** literature evidence to support the top 100 microRNAs for breast cancer | | | | | |
| --- | --- | --- | --- | --- | --- |
| **microRNA** | | **Rank** | **Supporting evidence** | **References** | |
| hsa-miR-429 | | 1 | Enforced expression of the miR-200 family (miR-200a, miR-200b, miR-200c, miR-141 and miR-429 and miR-205) alone was sufficient to prevent TGF-beta-induced EMT. These microRNAs cooperatively regulate expression of the E-cadherin transcriptional repressors ZEB1and SIP1, factors previously implicated in EMT and tumor metastasis. Inhibition of the microRNAs was sufficient to induce EMT in a process requiring upregulation of ZEB1 and/or SIP1. Conversely, ectopic expression of these microRNAs in mesenchymal cells initiated mesenchymal to epithelial transition (MET). Expression of these microRNAs was found to be lost in invasive breast cancer cell lines with mesenchymal phenotype and in regions of metaplastic breast cancer specimens lacking E-cadherin. | Gregory et al., 2008 | |
|  | |  |  |  | |
| hsa-miR-141 | | 2 | Enforced expression of the miR-200 family (miR-200a, miR-200b, miR-200c, miR-141 and miR-429 and miR-205) alone was sufficient to prevent TGF-beta-induced EMT. These microRNAs cooperatively regulate expression of the E-cadherin transcriptional repressors ZEB1and SIP1, factors previously implicated in EMT and tumor metastasis. Inhibition of the microRNAs was sufficient to induce EMT in a process requiring upregulation of ZEB1 and/or SIP1. Conversely, ectopic expression of these microRNAs in mesenchymal cells initiated mesenchymal to epithelial transition (MET). Expression of these microRNAs was found to be lost in invasive breast cancer cell lines with mesenchymal phenotype and in regions of metaplastic breast cancer specimens lacking E-cadherin. | Gregory et al., 2008 | |
|  | |  |  |  | |
| hsa-miR-200a | | 3 | Enforced expression of the miR-200 family (miR-200a, miR-200b, miR-200c, miR-141 and miR-429 and miR-205) alone was sufficient to prevent TGF-beta-induced EMT. These microRNAs cooperatively regulate expression of the E-cadherin transcriptional repressors ZEB1and SIP1, factors previously implicated in EMT and tumor metastasis. Inhibition of the microRNAs was sufficient to induce EMT in a process requiring upregulation of ZEB1 and/or SIP1. Conversely, ectopic expression of these microRNAs in mesenchymal cells initiated mesenchymal to epithelial transition (MET). Expression of these microRNAs was found to be lost in invasive breast cancer cell lines with mesenchymal phenotype and in regions of metaplastic breast cancer specimens lacking E-cadherin. | Gregory et al., 2008 | |
|  | |  |  |  | |
| hsa-miR-200b | | 4 | Enforced expression of the miR-200 family (miR-200a, miR-200b, miR-200c, miR-141 and miR-429 and miR-205) alone was sufficient to prevent TGF-beta-induced EMT. These microRNAs cooperatively regulate expression of the E-cadherin transcriptional repressors ZEB1and SIP1, factors previously implicated in EMT and tumor metastasis. Inhibition of the microRNAs was sufficient to induce EMT in a process requiring upregulation of ZEB1 and/or SIP1. Conversely, ectopic expression of these microRNAs in mesenchymal cells initiated mesenchymal to epithelial transition (MET). Expression of these microRNAs was found to be lost in invasive breast cancer cell lines with mesenchymal phenotype and in regions of metaplastic breast cancer specimens lacking E-cadherin. | Gregory et al., 2008 | |
|  | |  |  |  | |
| hsa-miR-29c | | 5 | Up-regulated greater than twofold in breast cancer compared with normal adjacent tumor tissues. | Yan et al., 2008 | |
|  | |  |  |  | |
| hsa-miR-196a | | 6 | Differentially expressed between breast carcinoma and normal breast tissue. | Iorio et al., 2005 | |
|  | |  |  |  | |
| hsa-miR-29b | | 8 | Volinia et al. found miR-29b was commonly up-regulated microRNA in 3 or more types of solid cancers. Yan et al.found it was Differentially expressed between breast carcinoma and normal breast tissue. | Volinia et al., 2006;  Yan et al., 2008 | |
|  | |  |  |  | |
| hsa-miR-20a | | 10 | Levels of the miR-17-5p/miR-20a miRNA cluster were inversely correlated to cyclin D1 abundance in human breast tumors and cell lines. MiR-17/20 suppressed breast cancer cell proliferation and tumor colony formation by negatively regulating cyclin D1 translation, thereby inhibiting serum-induced S phase entry. The cell cycle effect of miR-17/20 was abrogated by cyclin D1 siRNA and in cyclin D1-deficient breast cancer cells. Mammary epithelial cell-targeted cyclin D1 expression induced miR-17-5p and miR-20a expression in vivo, and cyclin D1 bound the miR-17/20 cluster promoter regulatory region. In summary, these studies identify a novel cyclin D1/miR-17/20 regulatory feedback loop through which cyclin D1 induces miR-17-5p/miR-20a. In turn, miR-17/20 limits the proliferative function of cyclin D1, thus linking expression of a specific miRNA cluster to the regulation of oncogenesis. | Yu et al.,2008 | |
|  | |  |  |  | |
| hsa-miR-200c | | 11 | Enforced expression of the miR-200 family(miR-200a, miR-200b, miR-200c, miR-141 and miR-429 and miR-205) alone was sufficient to prevent TGF-beta-induced EMT. These microRNAs cooperatively regulate expression of the E-cadherin transcriptional repressors ZEB1and SIP1, factors previously implicated in EMT and tumor metastasis. Inhibition of the microRNAs was sufficient to induce EMT in a process requiring upregulation of ZEB1 and/or SIP1. Conversely, ectopic expression of these microRNAs in mesenchymal cells initiated mesenchymal to epithelial transition (MET). Expression of these microRNAs was found to be lost in invasive breast cancer cell lines with mesenchymal phenotype and in regions of metaplastic breast cancer specimens lacking E-cadherin. | Gregory et al., 2008 | |
|  | |  |  |  | |
| hsa-miR-7 | | 23 | Reddy et al found that miR-7 inhibits p21-activated kinase 1 (Pak1) expression, a widely up-regulated signaling kinase in multiple human cancers and the miR-7 introduction inhibits the motility, invasiveness, anchorage-independent growth, and tumorigenic potential of highly invasive breast cancer cells. Foekens et al linked miR-7 to breast cancer aggressiveness. In addition, Webster et al found that miR-7 down-regulates EGFR mRNA and protein expression in cancer cell lines (lung, breast, and glioblastoma) via two of the three sites, inducing cell cycle arrest and cell death and that miR-7 attenuated activation of protein kinase B (Akt) and extracellular signal-regulated kinase 1/2, two critical effectors of EGFR signaling, in different cancer cell lines. | Reddy et al., 2008;  Foekens et al., 2008;  Webster et al., 2009 | |
|  | |  |  |  | |
| hsa-miR-335 | | 25 | miR-335 was observed to be down-regulated greater than twofold in breast cancer. | Yan et al., 2008 | |
|  | |  |  |  | |
| hsa-miR-125b | | 27 | miR-125b is down-modulated in breast cancer and locates at chromosome 11q23-24, one of the regions most frequently deleted in breast, ovarian, and lung tumors. The recognition of a bona fide tumor suppressor gene located at 11q23-24 involved in the pathogenesis of human breast cancer is still lacking. The miR-125b gene establishes itself as an important candidate for this role. Overexpression of miR-125a or miR-125b reduced ERBB2 and ERBB3 at both the transcript and protein level in these cells, leading to reduced ERK1/2 and AKT signaling. Functionally, miR-125a or miR-125b-overexpressing SKBR3 cells displayed diminished plating and anchorage-dependent growth in addition to markedly reduced cell migration and invasion capacities. | Iorio et al., 2005;  Scott et al., 2007 | |
|  | |  |  |  | |
| hsa-miR-98 | | 28 | miR-98 was observed to be down-regulated greater than twofold in breast cancer. | Yan et al., 2008 | |
|  | |  |  |  | |
| hsa-miR-127-5p | | 33 | miR-127 is usually expressed as part of a miRNA cluster in normal cells but not in cancer cells, suggesting that it is subject to epigenetic silencing. In addition, the proto-oncogene BCL6, a potential target of miR-127, was translationally downregulated. These results suggest that DNA demethylation and histone deacetylase inhibition can activate expression of miRNAs that may act as tumor suppressors. | Yan et al., 2008;  Saito et al., 2006 | |
|  | |  |  |  | |
| hsa-miR-31 | | 42 | miR-31 was observed to be down-regulated greater than twofold in breast cancer. | Yan et al., 2008 | |
|  | |  |  |  | |
| hsa-miR-17 | | 46 | Hossain et al found that miR-17-5p Regulates breast cancer Cell Proliferation by Inhibiting Translation of AIB1 mRNA. In addition, levels of the miR-17-5p/miR-20a miRNA cluster were inversely correlated to cyclin D1 abundance in human breast tumors and cell lines. MiR-17/20 suppressed breast cancer cell proliferation and tumor colony formation by negatively regulating cyclin D1 translation, thereby inhibiting serum-induced S phase entry. The cell cycle effect of miR-17/20 was abrogated by cyclin D1 siRNA and in cyclin D1-deficient breast cancer cells. Mammary epithelial cell-targeted cyclin D1 expression induced miR-17-5p and miR-20a expression in vivo, and cyclin D1 bound the miR-17/20 cluster promoter regulatory region. In summary, these studies identify a novel cyclin D1/miR-17/20 regulatory feedback loop through which cyclin D1 induces miR-17-5p/miR-20a. In turn, miR-17/20 limits the proliferative function of cyclin D1, thus linking expression of a specific miRNA cluster to the regulation of oncogenesis. | Hossain et al., 2006;  Volinia et al.,2006;  Yu et al., 2008 | |
|  | |  |  |  | |
| hsa-let-7f | | 47 | Iorio et al. found let-7f was Differentially expressed between breast carcinoma and normal breast tissue. Jiang et al found its precursors was significantly different expression. In addition, Yan et al. found it was observed to be up-regulated greater than twofold in breast cancer compared with normal adjacent tumor tissues. | Iorio et al., 2005;  Jiang et al., 2005;  Yan et al., 2008 | |
|  | |  |  |  | |
| hsa-let-7i | | 50 | Differentially expressed between breast carcinoma and normal breast tissue. | Iorio et al., 2005 | |
|  | |  |  |  | |
| hsa-miR-221 | | 56 | Zhao et al. found that miR-221 and miR-222 directly interact with the 3'-untranslated region of ERalpha. Ectopic expression of miR-221 and miR-222 in MCF-7 and T47D cells resulted in a decrease in expression of ERalpha protein but not mRNA, whereas knockdown of miR-221 and miR-222 partially restored ERalpha in ERalpha protein-negative/mRNA-positive cells. Notably, miR-221- and/or miR-222-transfected MCF-7 and T47D cells became resistant to tamoxifen compared with vector-treated cells. Knockdown of miR-221 and/or miR-222 sensitized MDA-MB-468 cells to tamoxifen-induced cell growth arrest and apoptosis. These findings indicate that miR-221 and miR-222 play a significant role in the regulation of ERalpha expression at the protein level and could be potential targets for restoring ERalpha expression and responding to antiestrogen therapy in a subset of breast cancers. Miller found that the protein level of the cell cycle inhibitor p27 was reduced by 50% in OHT(R) cells and by 28-50% in miR-221/222-overexpressing MCF-7 cells. Furthermore, overexpression of p27 in the resistant OHT(R) cells caused enhanced cell death when exposed to tamoxifen. | Zhao et al., 2008;  Miller et al., 2008; | |
|  | |  |  |  | |
| hsa-miR-16-1 | | 57 | Zhang et al. showed a copy number loss of the regions containing mir-15a and mir-16-1 in 23.9% of ovarian and 24.7% of breast cancers. | Zhang et al., 2006 | |
|  | |  |  |  | |
| hsa-miR-202 | | 64 | Differentially expressed between breast carcinoma and normal breast tissue. | Iorio et al., 2005 | |
|  | |  |  |  | |
| hsa-miR-373 | | 67 | miR-373 and miR-520c stimulated cancer cell migration and invasion in vitro and in vivo. Mechanistically, the migration phenotype of miR-373 and miR-520c can be explained by suppression of CD44. We found significant upregulation of miR-373 in clinical breast cancer metastasis samples that correlated inversely with CD44 expression. | Huang et al., 2008 | |
|  | |  |  |  | |
| hsa-let-7a | | 68 | Increased let-7 paralleled reduced H-RAS and HMGA2, known let-7 targets. Silencing H-RAS in a breast tumor-initiating cells(BTIC)- enriched cell line reduced self renewal but had no effect on differentiation, while silencing HMGA2 enhanced differentiation but did not affect self renewal. Therefore let-7 regulates multiple BT-IC stem cell-like properties by silencing more than one target. | Yu et al., 2008;  Iorio et al., 2005 | |
|  | |  |  |  | |
| hsa-miR-497 | | 73 | miR-497 was observed to be down-regulated greater than twofold in breast cancer. | Yan et al., 2008 | |
|  | |  |  |  | |
| hsa-let-7d | | 79 | Differentially expressed between breast carcinoma and normal breast tissue. | Iorio et al., 2005 | |
|  | |  |  |  | |
| hsa-miR-510 | | 88 | A putative tumor suppressor PDEF protein expression is often lost during progression to a more invasive phenotype. Findlay et al. identifies two microRNAs-miR-204 and miR-510 that directly act on and repress PDEF mRNA translation, leading to the loss of PDEF protein expression and the gain of phenotypes associated with invasive cells. In addition, these miRNAs are elevated in human breast tumor samples. Together, these data describe a mechanism of regulation that explains, for the first time, the lack of correlation between PDEF mRNA and protein levels, providing insight into the underexplored role of posttranscriptional regulation and how this contributes to dysregulated protein expression in cancer. These observations have critical implications for therapeutically targeting miRNAs that contribute to cancer progression. | Findlay et al., 2008 | |
|  |  | |  | |  |
| hsa-miR-18a | 93 | | Real-time PCR showed that mir-18a and mir-195 were highly expressed in MCF-7 cells. | | Zhang et al., 2009 |
|  |  | |  | |  |
| hsa-miR-146b-5p | | 96 | Bhaumik *et al*.demonstrated that microRNA-146a and microRNA-146b (miR-146a/b) when expressed in the highly metastatic human breast cancer cell line MDA-MB-231 function to negatively regulate NF-kappaB activity. Functionally, miR-146a/b-expressing MDA-MB-231 cells showed markedly impaired invasion and migration capacity relative to control cells. These findings implicate miR-146a/b as a negative regulator of constitutive NF-kappaB activity in a breast cancer setting and suggest that modulating miR-146a/b levels has therapeutic potential to suppress breast cancer metastases. | Bhaumik et al., 2008 | |
|  | |  |  |  | |
| hsa-miR-155 | | 100 | Transforming growth factor beta (TGF-beta) signaling facilitates metastasis in advanced malignancy. Among upregulated miRNAs, miR-155 was the most significantly elevated miRNA. TGF-beta induces miR-155 expression and promoter activity through Smad4. The knockdown of miR-155 suppressed TGF-beta-induced epithelial-mesenchymal transition (EMT) and tight junction dissolution, as well as cell migration and invasion. Further, the ectopic expression of miR-155 reduced RhoA protein and disrupted tight junction formation. Reintroducing RhoA cDNA without the 3' untranslated region largely reversed the phenotype induced by miR-155 and TGF-beta. In addition, elevated levels of miR-155 were frequently detected in invasive breast cancer tissues. These data suggest that miR-155 may play an important role in TGF-beta-induced EMT and cell migration and invasion by targeting RhoA and indicate that it is a potential therapeutic target for breast cancer intervention. | Kong et al., 2008 | |

**References**

Bhaumik D, Scott GK, Schokrpur S, Patil CK, Campisi J, Benz CC (2008) Expression of microRNA-146 suppresses NF-kappaB activity with reduction of metastatic potential in breast cancer cells. *Oncogene*.

Findlay VJ, Turner DP, Moussa O, Watson DK (2008) MicroRNA-mediated inhibition of prostate-derived Ets factor messenger RNA translation affects prostate-derived Ets factor regulatory networks in human breast cancer. *Cancer Res* **68:** 8499-8506.

Foekens JA, Sieuwerts AM, Smid M, Look MP, de Weerd V, Boersma AW, Klijn JG, Wiemer EA, Martens JW (2008) Four miRNAs associated with aggressiveness of lymph node-negative, estrogen receptor-positive human breast cancer. *Proc Natl Acad Sci U S A* **105:** 13021-13026.

Gregory PA, Bert AG, Paterson EL, Barry SC, Tsykin A, Farshid G, Vadas MA, Khew-Goodall Y, Goodall GJ (2008) The miR-200 family and miR-205 regulate epithelial to mesenchymal transition by targeting ZEB1 and SIP1. *Nat Cell Biol* **10:** 593-601.

Hossain A, Kuo MT, Saunders GF (2006) Mir-17-5p regulates breast cancer cell proliferation by inhibiting translation of AIB1 mRNA. *Mol Cell Biol* **26:** 8191-8201.

Huang Q, Gumireddy K, Schrier M, le Sage C, Nagel R, Nair S, Egan DA, Li A, Huang G, Klein-Szanto AJ, Gimotty PA, Katsaros D, Coukos G, Zhang L, Pure E, Agami R (2008) The microRNAs miR-373 and miR-520c promote tumour invasion and metastasis. *Nat Cell Biol* **10:** 202-210.

Iorio MV, Ferracin M, Liu CG, Veronese A, Spizzo R, Sabbioni S, Magri E, Pedriali M, Fabbri M, Campiglio M, Menard S, Palazzo JP, Rosenberg A, Musiani P, Volinia S, Nenci I, Calin GA, Querzoli P, Negrini M, Croce CM (2005) MicroRNA gene expression deregulation in human breast cancer. *Cancer Res* **65:** 7065-7070.

Jiang J, Lee EJ, Gusev Y, Schmittgen TD (2005) Real-time expression profiling of microRNA precursors in human cancer cell lines. *Nucleic Acids Res* **33:** 5394-5403.

Kong W, Yang H, He L, Zhao JJ, Coppola D, Dalton WS, Cheng JQ (2008) MicroRNA-155 is regulated by the transforming growth factor beta/Smad pathway and contributes to epithelial cell plasticity by targeting RhoA. *Mol Cell Biol* **28:** 6773-6784.

Miller TE, Ghoshal K, Ramaswamy B, Roy S, Datta J, Shapiro CL, Jacob S, Majumder S (2008) MicroRNA-221/222 confers tamoxifen resistance in breast cancer by targeting p27Kip1. *J Biol Chem* **283:** 29897-29903.

Reddy SD, Ohshiro K, Rayala SK, Kumar R (2008) MicroRNA-7, a homeobox D10 target, inhibits p21-activated kinase 1 and regulates its functions. *Cancer Res* **68:** 8195-8200.

Saito Y, Liang G, Egger G, Friedman JM, Chuang JC, Coetzee GA, Jones PA (2006) Specific activation of microRNA-127 with downregulation of the proto-oncogene BCL6 by chromatin-modifying drugs in human cancer cells. *Cancer Cell* **9:** 435-443.

Scott GK, Goga A, Bhaumik D, Berger CE, Sullivan CS, Benz CC (2007) Coordinate suppression of ERBB2 and ERBB3 by enforced expression of micro-RNA miR-125a or miR-125b. *J Biol Chem* **282:** 1479-1486.

Volinia S, Calin GA, Liu CG, Ambs S, Cimmino A, Petrocca F, Visone R, Iorio M, Roldo C, Ferracin M, Prueitt RL, Yanaihara N, Lanza G, Scarpa A, Vecchione A, Negrini M, Harris CC, Croce CM (2006) A microRNA expression signature of human solid tumors defines cancer gene targets. *Proc Natl Acad Sci U S A* **103:** 2257-2261.

Webster RJ, Giles KM, Price KJ, Zhang PM, Mattick JS, Leedman PJ (2009) Regulation of epidermal growth factor receptor signaling in human cancer cells by microRNA-7. *J Biol Chem* **284:** 5731-5741.

Yan LX, Huang XF, Shao Q, Huang MY, Deng L, Wu QL, Zeng YX, Shao JY (2008) MicroRNA miR-21 overexpression in human breast cancer is associated with advanced clinical stage, lymph node metastasis and patient poor prognosis. *RNA* **14:** 2348-2360.

Yu F, Yao H, Zhu P, Zhang X, Pan Q, Gong C, Huang Y, Hu X, Su F, Lieberman J, Song E (2007) let-7 regulates self renewal and tumorigenicity of breast cancer cells. *Cell* **131:** 1109-1123.

Yu Z, Wang C, Wang M, Li Z, Casimiro MC, Liu M, Wu K, Whittle J, Ju X, Hyslop T, McCue P, Pestell RG (2008) A cyclin D1/microRNA 17/20 regulatory feedback loop in control of breast cancer cell proliferation. *J Cell Biol* **182:** 509-517.

Zhang, H., Su, S.B., Zhou, Q.M. and Lu, Y.Y. (2009) [Differential expression profiles of microRNAs between breast cancer cells and mammary epithelial cells.], Ai Zheng, 28, 493-499.

Zhang L, Huang J, Yang N, Greshock J, Megraw MS, Giannakakis A, Liang S, Naylor TL, Barchetti A, Ward MR, Yao G, Medina A, O'Brien-Jenkins A, Katsaros D, Hatzigeorgiou A, Gimotty PA, Weber BL, Coukos G (2006) microRNAs exhibit high frequency genomic alterations in human cancer. *Proc Natl Acad Sci U S A* **103:** 9136-9141.

Zhao JJ, Lin J, Yang H, Kong W, He L, Ma X, Coppola D, Cheng JQ (2008) MicroRNA-221/222 negatively regulates estrogen receptor alpha and is associated with tamoxifen resistance in breast cancer. *J Biol Chem* **283:** 31079-31086.
